# VITCOMIC: visualization tool for taxonomic compositions of microbial communities based on 16S rRNA gene sequences

**DOI:** 10.1186/1471-2105-11-332

**Published:** 2010-06-18

**Authors:** Hiroshi Mori, Fumito Maruyama, Ken Kurokawa

**Affiliations:** 1 Department of Biological Information, Graduate School of Bioscience and Biotechnology, Tokyo Institute of Technology, 4259 B-36, Nagatsuta-cho, Midori-ku, Yokohama 226-8501, Japan

## Abstract

**Background:**

Understanding the community structure of microbes is typically accomplished by sequencing 16S ribosomal RNA (16S rRNA) genes. These community data can be represented by constructing a phylogenetic tree and comparing it with other samples using statistical methods. However, owing to high computational complexity, these methods are insufficient to effectively analyze the millions of sequences produced by new sequencing technologies such as pyrosequencing.

**Results:**

We introduce a web tool named VITCOMIC (**VI**sualization tool for **T**axonomic **CO**mpositions of **MI**crobial **C**ommunity) that can analyze millions of bacterial 16S rRNA gene sequences and calculate the overall taxonomic composition for a microbial community. The 16S rRNA gene sequences of genome-sequenced strains are used as references to identify the nearest relative of each sample sequence. With this information, VITCOMIC plots all sequences in a single figure and indicates relative evolutionary distances.

**Conclusions:**

VITCOMIC yields a clear representation of the overall taxonomic composition of each sample and facilitates an intuitive understanding of differences in community structure between samples. VITCOMIC is freely available at http://mg.bio.titech.ac.jp/vitcomic/.

## Background

The number of sequenced bacterial genomes has increased rapidly and now exceeds 1,000 [1]; however, we have little information regarding environmental microbes, largely because the majority of them are unculturable [2]. The taxonomic composition of a microbial community can provide important clues to better understand its structure and ecology [3]. Analysis using 16S rRNA genes is a frequently used method to obtain the taxonomic composition of a microbial community [4,5]. Features of 16S rRNA genes include essentiality for all *Bacteria *and *Archaea*, mosaic structures of highly conserved regions and variable regions [6,7], and little possibility for horizontal gene transfer [8]. Moreover, the availability of numerous tools and databases specific for the 16S rRNA genes has potentiated taxonomic analyses [9-12].

Ultra-deep sequencing of microbial communities using a massively parallel pyrosequencer has recently uncovered relatively rare species in communities [5,13-15]. However, the enormous amounts of sequencing data produced by recent pyrosequencing studies are difficult to effectively analyze using existing computational tools (Additional file 1) [16]. For example, the overall taxonomic composition of each sample is traditionally presented graphically in phylogenetic trees [9,17]. However, graphical representation and comparison of overall taxonomic compositions for pyrosequencing data is difficult due to the high computational complexity involved in constructing multiple alignments and phylogenetic trees from millions of sequences [16,18]. Therefore, researchers tend to use a compressed representation of taxonomic composition such as a bar graph or pie chart of the phylum-level composition. Unfortunately, these compressed representations of overall taxonomic composition can be difficult to represent differences among microbial communities, especially differences attributable to minority taxa [19].

To address deficiencies in the analysis of taxonomic compositions of microbial communities, we developed a rapid visualization tool, named VITCOMIC, that presents overall taxonomic compositions based on large datasets of 16S rRNA genes from microbial communities. VITCOMIC can facilitate intuitive understanding of microbial communities and compare taxonomic compositions between communities.

## Implementation

### Creation of a reference 16S rRNA gene database and their distance matrix

The reference 16S rRNA gene sequence database was constructed using 16S rRNA gene sequences from genome-sequenced strains. These data are suitable as reference data because they are accurate and have well-defined taxonomic information. Genomic sequences of *Bacteria *and *Archaea *were obtained from the NCBI Genome Database [20] in September 2009. The 16S rRNA genes of each strain were detected using RNAmmer [21]. One 16S rRNA gene was randomly sampled per species because there are only small sequence differences among 16S rRNA genes within the same genome and the same species [22,23]. A total of 601 16S rRNA gene sequences from 601 species of *Bacteria *and *Archaea *were obtained. To calculate phylogenetic distances among them, all sequences were aligned using MAFFT 6.713 with default parameters [24]. After constructing multiple alignments, genetic distances between sequences with Kimura's two-parameter model of base substitution [25] were calculated using the dnadist program in PHYLIP 3.69 [26]. The phylogenetic tree was constructed using the neighbor-joining method in the neighbor program in PHYLIP 3.69. The phylum-level taxonomy of the species was obtained from the NCBI Taxonomy Database [27].

### Sample data for testing VITCOMIC

We used human gut microbiome data from Turnbaugh et al. [15] to test VITCOMIC. In their study, each individual was categorized as obese, lean, or overweight using body mass index. DNA was extracted from the feces of each individual, and the V2 variable regions of 16S rRNA genes were PCR amplified prior to pyrosequencing using a 454 GS FLX system [28]. We used the sequences from obese and lean individuals. The obese sample consisted of 704,369 sequences from 196 individuals; the lean sample consisted of 291,993 sequences from 61 individuals.

### Inference of a nearest relative for each sequence

**Figure 1:**
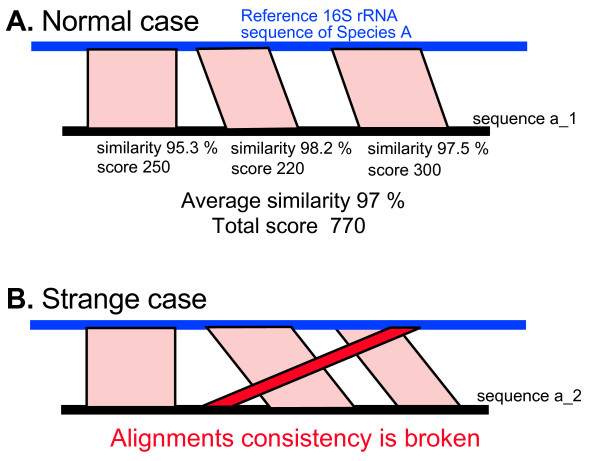
**Calculation of total BLAST scores and average similarities**. **(A) **A diagram of calculated total BLAST scores and average similarities between the sample and database sequences. **(B) **An example of a collapse of the alignment consistency caused by a false alignment.

Using the human gut microbiome data, we conducted BLASTN searches against the reference 16S rRNA gene database to determine a nearest relative for each sample sequence. The nearest relative is the evolutionally nearest database sequence of each sample sequence. In general, the reference sequence with the highest BLAST score is chosen as the nearest relative in sequence analyses [29]. However, because the 16S rRNA gene has mosaic structures of highly conserved regions and variable regions [6,7], the alignments created by BLAST are often divided by variable regions [30]. In this case, the BLAST score is calculated for each divided alignment, because overall BLAST scores between the sample and database sequences cannot be calculated using only the highest score alignment. To overcome this problem, we calculated a total BLAST score for alignments derived from the same pair of sample and database sequences. As illustrated in Figure 1A, the total BLAST score is calculated by summing BLAST scores of three divided alignments from the same pair of sample and database sequences (250 + 220 + 300 = 770). To identify the nearest relative of the sample sequence, the total BLAST score is calculated against each database sequence. Upon comparison with the total BLAST scores between database sequences, the database sequence with the highest total BLAST score is adopted as the nearest relative of the sample sequence.

Alignments less than 50 bp were excluded to avoid inaccurate alignments. Because variable regions are nearly neutral, false alignments between a variable region and a conserved region or other variable regions are sometimes constructed and included in calculations of total BLAST scores (Figure 1B). To calculate total BLAST scores, it is necessary to develop the function "alignments consistency check". The alignments consistency check detects false alignment using information on positions of aligned regions of the sample sequence and matched database sequence. Normally, the order of aligned regions of the sample sequence is consistent with that of the matched database sequence (Figure 1A). On the other hand, most pairs of sequences that contain false alignments are not consistent with respect to the order of aligned regions (Figure 1B). The alignments consistency check detects collapses of these consistencies and excludes these pairs of sample and database sequences in the target calculation of total BLAST scores.

### Graphical representation of the taxonomic composition of the sample

**Figure 2:**
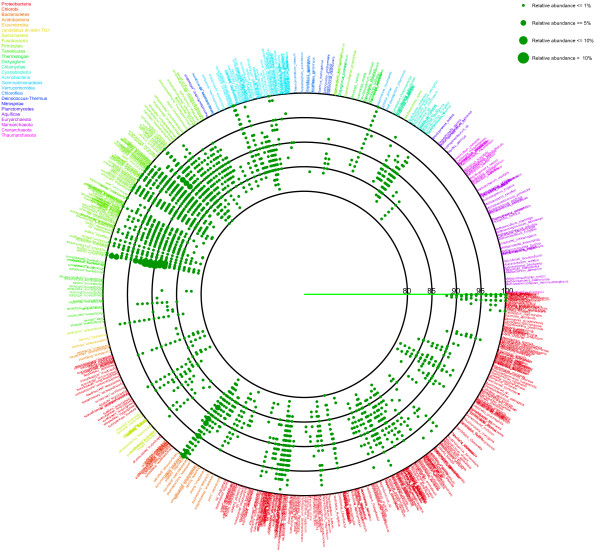
**Mapping result for the human gut sample from obese individuals**.

**Figure 3:**
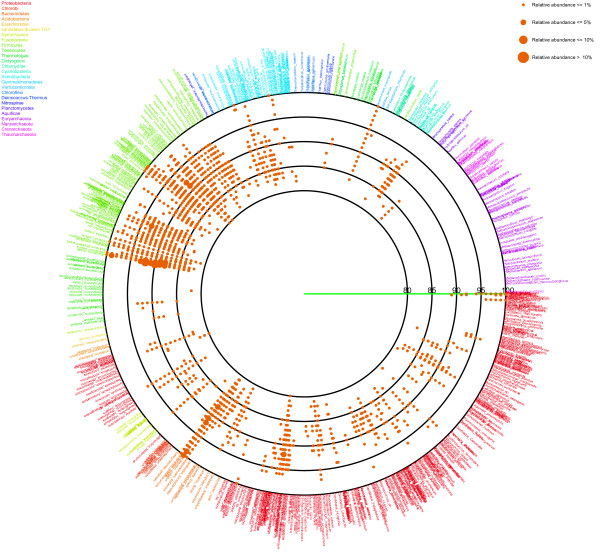
**Mapping result for the human gut sample from lean individuals**.

**Figure 4:**
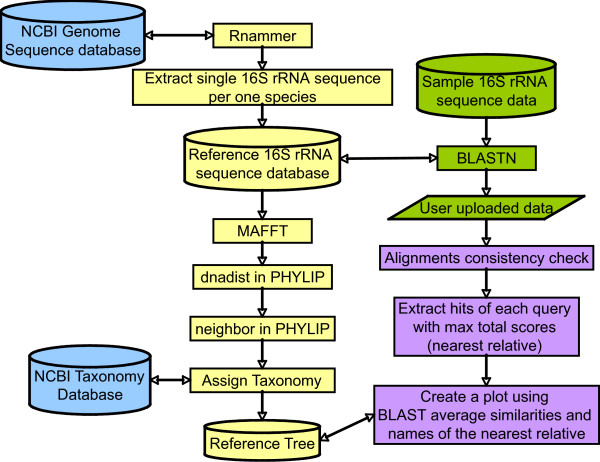
**VITCOMIC flow chart**.

After determining the nearest relative of each sample sequence, an average similarity between the sample sequence and the nearest relative was calculated from each set of alignments (Figure 1A). Information on the nearest relative and the average similarity is represented as a circle plot (Figures 2 and 3). In the figures, each species name in the reference 16S rRNA gene database is placed outside of the most lateral circle with ordered phylogenetic relatedness. Physical distances between nearest species in the plot indicate genetic distances of 16S rRNA genes between them. The font color for each species name corresponds to its phylum name. Large circles indicate boundaries of BLAST average similarities (inner most circle starting at 80%, followed by 85, 90, 95 and 100% similarity of the database sequence). Small colored dots represent average similarities of each sequence against the nearest relative species. The size of these dots indicates relative abundance of sequences in the sample. The figure produced by VITCOMIC contains four categories of dot size that indicate the relative abundance of the sample sequence: smallest dot < 1%; second smallest dot < 5%; third smallest dot < 10% (largest dot in Figures 2 and 3); and the largest dot > 10%. The results are outputted as a Postscript file that can be viewed at high resolution. The overall workflow of VITCOMIC is described in Figure 4. The input file of VITCOMIC is basically a result file of BLAST against our reference 16S rRNA gene sequence database. Our reference database can be downloaded from the VITCOMIC web site http://mg.bio.titech.ac.jp/vitcomic/. When analyzing small amounts of data (less than 100,000 sequences), the multi-FASTA file before BLAST is accepted as the input file. The VITCOMIC web site contains detailed instructions for users.

### Comparison of taxonomic compositions between samples

**Figure 5:**
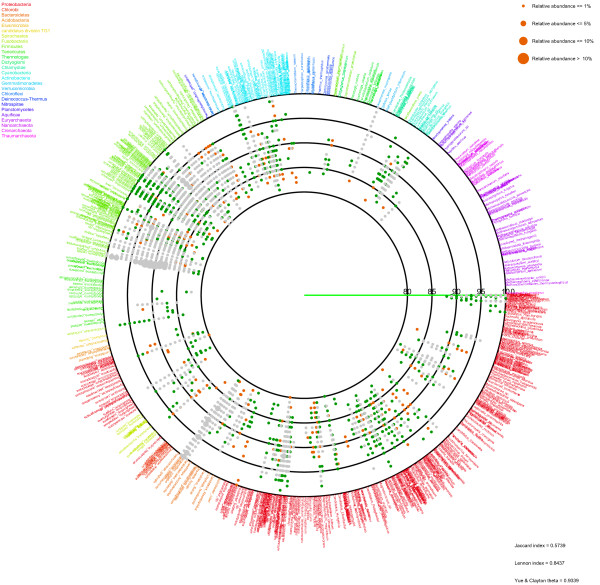
**Merged results for the obese and lean human gut samples**.

To compare taxonomic compositions between samples, VITCOMIC clusters sample sequences using single-linkage clustering with 99% similarity as follows. When a sample sequence is assigned to a reference species according to a certain average similarity as described above, VITCOMIC rounds down the average similarity to the integer. If the rounded average similarity and the matched reference species are identical between sample sequences, VITCOMIC clusters these sequences together. For example, one sequence was assigned to *Bacillus subtilis *with 98.8% average similarity, whereas another sequence was assigned to *B. subtilis *with 98.1% average similarity; VITCOMIC clusters these sequences in the *B. subtilis *98% cluster. After applying this single-linkage clustering based on reference sequences with 99% similarity to each sample, VITCOMIC compares the clustering results to identify common clusters between samples. When the cluster that is assigned to the same reference species and sequence similarity exists both of the samples, the cluster is designated as a common cluster between samples. Using information on common clusters between samples, VITCOMIC creates a merged plot the one shown in Figure 5. Gray dots indicate common clusters between the obese and lean samples, green dots indicate specific clusters of the obese samples, and orange dots indicate specific clusters of the lean samples. For statistical comparison of taxonomic compositions between samples, VITCOMIC calculates three types of similarity indices for taxonomic compositions between samples using the clustering result (Jaccard index, Lennon index, and Yue and Clayton theta index) [31]. These indices are shown in the lower-right portion of the merged plot (Figure 5).

## Results

Using VITCOMIC, the overall taxonomic compositions of both the obese and lean samples could be clearly visualized (Figure 2 = obese; Figure 3 = lean). Large colored dots indicate relatively abundant taxa in each sample (relative abundance > 1%). These large colored dots are distributed almost identically between obese and lean samples and are located at related species of *Clostridium*, *Eubacterim*, and *Bacteroides*. These taxa are the abundant in the normal human gut microbiome [32]. Small dots that are located at the most lateral circle indicate closely related strains of the genome-sequenced strains. These strains are *Escherichia coli *and *Proteus mirabilis *in *Proteobacteria*, *Enterococcus faecalis *and the group of *Lactobacillus *in *Firmicutes*, groups of *Bifidobacterium *and *Propionibacterium *in *Actinobacteria*, and *Akkermansia muciniphila *in *Verrucomicrobia*. It is well established that some of these strains inhabit the human gut, whereas others do not [33-39]. In Figures 2 and 3, several dots are distributed on the 80-90% lines, indicating that several taxa distantly related to genome-sequenced strains inhabit the human gut. These results were consistent with the study of Turnbaugh et al. [15].

**Figure 6:**
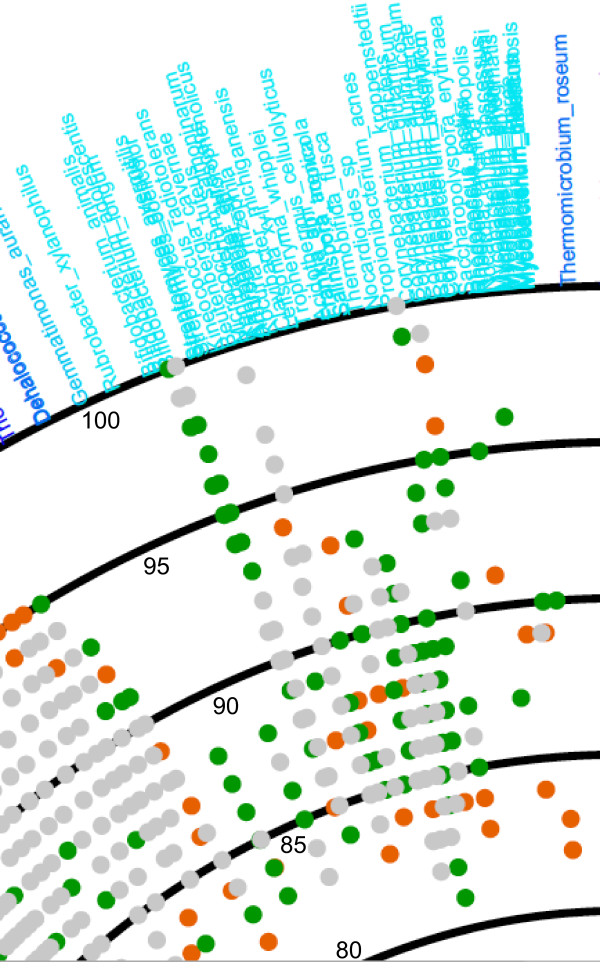
**High-resolution view of the region containing the phylum *Actinobacteria *in Figure 5**.

Differences between the obese and lean samples are clearly evident in Figure 5, which was created by the comparing function of VITCOMIC. Gray dots indicate common taxa between the obese and lean samples; green dots indicate specific taxa of the obese samples, and orange dots indicate specific taxa of the lean samples. The majority of taxa appear to be common between obese and lean samples, although certain taxa could be specific to the obese or lean sample (for example, the phylum *Actinobacteria *in the obese sample as described in the study of Turnbaugh et al. [15]). Figure 6 presents a higher resolution view of the region related to *Actibobacteria *in Figure 5.

## Discussion

VITCOMIC can easily visualize overall taxonomic compositions of large amounts of 16S rRNA gene-based community analysis data. Traditional visualization methods by constructing phylogenetic trees require a lot of computation time when analyzing large amounts of data [16]. Even if researchers are able to construct a phylogenetic tree, the tree itself can be difficult to analyze because it may contain too many branches [29]. By contrast, taxonomic assignments based on BLAST are fast and can be highly parallelized [40]. Although several highly accurate taxonomic assignment tools have been developed [41,42], the accuracy of BLAST-based taxonomic assignments is also well validated [29,43]. In addition, calculations of total BLAST scores and applications of the alignments consistency check improve the accuracy of the assignment, especially when long sequences are examined. Longer sequences containing more variable regions will generate a greater number of alignment divisions. The alignments consistency check may be necessary for the study using the pyrosequencer because recently developed pyrosequencer has improved the read length by over 400 bp [44]. Although the taxonomic assignment using only genome-sequenced species for the reference would not yield the best assignment compared with the assignment using larger database that contains uncultured bacteria [12,45], this provisional taxonomy provided by VITCOMIC is accurate enough for the visual comparisons of taxonomic composition between samples.

Compared with other tools, the most unique function of VITCOMIC is a simultaneous visualization and comparison of taxonomic compositions between samples (Additional file 1). Comparison of taxonomic compositions between samples from different microbial communities is an effective means to better understand similarities and differences between microbial communities [10]. However, the comparison of several microbial communities can be difficult given a large number of sequences [16]. VITCOMIC can simultaneously visualize large amounts of data by merging sequence data from several community analysis projects (Additional files 2, 3, and 4). Additional file 2 visualizes 139,356 16S rRNA gene sequences obtained from various soils [13]. Additional file 3 presents seawater microbial communities data derived from 452 different 16S rRNA gene surveys containing 11,144,358 sequences, which were obtained from the NCBI Sequence Read Archive [46]. Additional file 4 presents data for the human microbial communities derived from 60 different 16S rRNA gene surveys containing 4,363,040 sequences, which were obtained from NCBI Sequence Read Archive. Although detailed comparisons among samples from different microbial communities are difficult due to the large number of sequences and differing primers, VITCOMIC showed that overall taxonomic compositions and abundant taxa are distinctly different between environments.

VITCOMIC only uses the 16S rRNA gene sequences from 601 genome-sequenced bacteria as references. The reason why we selected the reference database from 601 species is the quality and quantity of the biological information. These sequences are derived from genome-sequenced species, from which we can generally obtain much information about ecophysiology (i.e., metabolic potentials, habitats, gene repertoires). Therefore, by adopting genome-sequenced species as the reference database, we can retrieve several biological information for each taxon inductively by analyzing the genomic information of the nearest genome-sequenced species from the 16S rRNA gene-targeted analysis. These features provide valuable initiative knowledge for a following metagenomic analysis. To address the increasing number of genome-sequenced species, the reference database of VITCOMIC will be updated periodically.

## Conclusions

Using a phylogenetic relationship with genome-sequenced strains, VITCOMIC clearly presents the overall taxonomic composition of 16S rRNA gene-based microbial community analysis data. VITCOMIC facilitates an intuitive understanding of differences in community structure between samples.

## Availability and requirements

• **Project name: **VITCOMIC

• **Project home page: **http://mg.bio.titech.ac.jp/vitcomic/

• **Operating system(s): **Platform independent

• **Programming language: **Perl

• **Other requirements: **None

• **License: **GNU GPL

• **Any restrictions to use by non-academics: **None

## Authors' contributions

HM and KK designed the study. HM developed the method and performed the analyses. FM and KK provided advise on method design and analyses. HM drafted the manuscript, and FM and KK critically revised it. All authors read and approved the final manuscript.

## Supplementary Material

Additional file 1 **Comparison of VITCOMIC's features relative to existing commonly used 16S rRNA gene analysis tools**. Click here for file

Additional file 2 **Mapping result for the soil microbial community analyses data**. The soil microbial community analyses data derived from 4 different soils that included 139,356 16S rRNA gene sequences [13]. Click here for file

Additional file 3 **Mapping result for the seawater microbial community analyses data**. The seawater microbial community analyses data derived from 452 experiments that included 11,144,358 sequences were obtained from the NCBI Sequence Read Archive on December 16, 2009. Click here for file

Additional file 4 **Mapping result for the human microbial community analyses data**. The human microbial community analyses data derived from 60 experiments that included 4,363,040 sequences were obtained from the NCBI Sequence Read Archive on December 16, 2009. Click here for file
