# Supplementary material for: VITCOMIC: visualization tool for taxonomic compositions of microbial communities based on 16S rRNA gene sequences
Source: BMC Bioinformatics. 2010 Jun 18;11:332. doi: 10.1186/1471-2105-11-332 (PMC2894824; doi:10.1186/1471-2105-11-332)
Supplement: Additional file 2 — Mapping result for the soil microbial community analyses data. The soil microbial community analyses data derived from 4 different soils that included 139,356 16S rRNA gene sequences [13]. [file 1471-2105-11-332-S2.PDF]

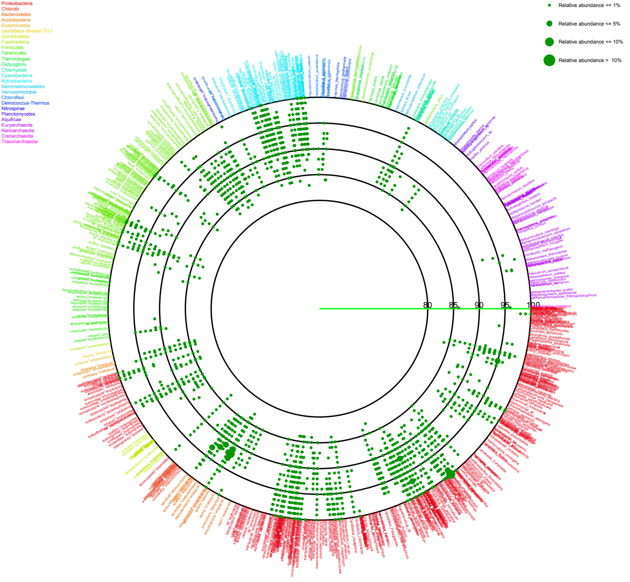
Proteobacteria
Chlorobi
Bacteroidetes
Acidobacteria
Elusimicrobia
candidatus division TG1
Spirochaetes
Fusobacteria
Firmicutes
Tenericutes
Thermotogae
Dictyoglomi
Chlamydiae
Cyanobacteria
Actinobacteria
Gemmatimonadetes
Verrucomicrobia
Chloroflexi
Deinococcus-Thermus
Nitrospirae
Planctomycetes
Aquificae
Euryarchaeota
Nanoarchaeota
Crenarchaeota
Thaumarchaeota
Relative abundance <= 1%
Relative abundance <= 5%
Relative abundance <= 10%
Relative abundance > 10%
80
85
90
95
100
