# Supplementary figures and images for: VITCOMIC: visualization tool for taxonomic compositions of microbial communities based on 16S rRNA gene sequences
Source: BMC Bioinformatics. 2010 Jun 18;11:332. doi: 10.1186/1471-2105-11-332 (PMC2894824; doi:10.1186/1471-2105-11-332)

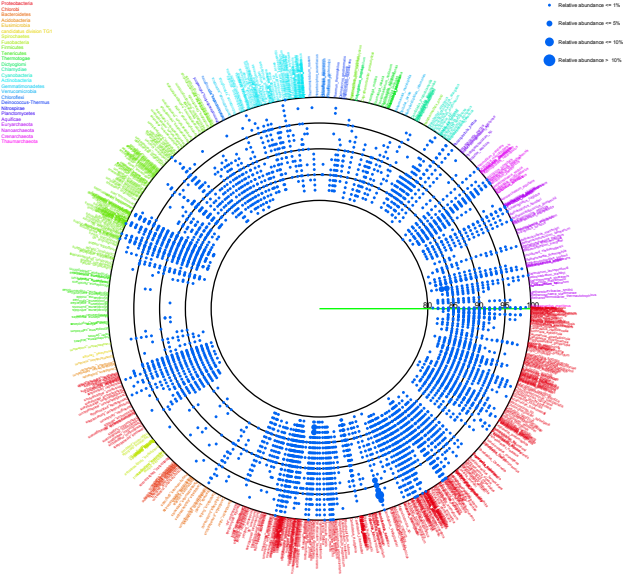

Supplement: Additional file 3 — Mapping result for the seawater microbial community analyses data. The seawater microbial community analyses data derived from 452 experiments that included 11,144,358 sequences were obtained from the NCBI Sequence Read Archive on December 16, 2009. [file 1471-2105-11-332-S3.PDF]

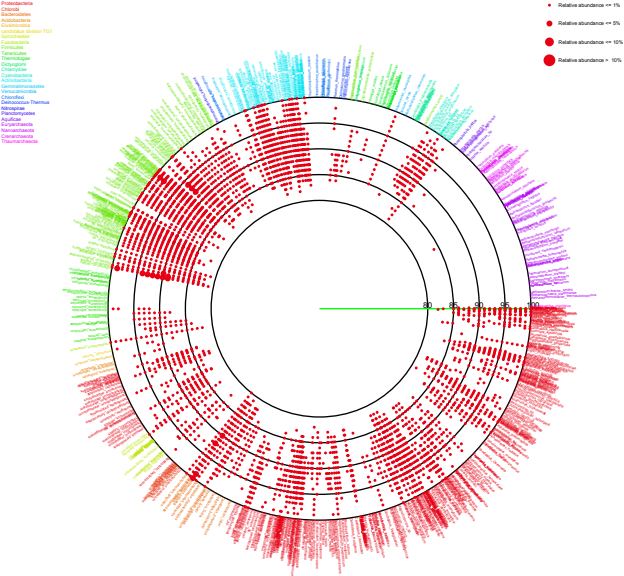

Supplement: Additional file 4 — Mapping result for the human microbial community analyses data. The human microbial community analyses data derived from 60 experiments that included 4,363,040 sequences were obtained from the NCBI Sequence Read Archive on December 16, 2009. [file 1471-2105-11-332-S4.PDF]
